# IL-39 promotes chronic graft-versus-host disease by increasing T and B Cell pathogenicity

**DOI:** 10.1186/s40164-022-00286-x

**Published:** 2022-06-02

**Authors:** Kangkang Lv, Bo Hu, Mingzhu Xu, Li Wan, Ziqi Jin, Mimi Xu, Yuanyuan Du, Kunpeng Ma, Quansheng Lv, Yang Xu, Lei Lei, Huanle Gong, Haiyan Liu, Depei Wu, Yuejun Liu

**Affiliations:** 1 National Clinical Research Center for Hematologic Diseases, Jiangsu Institute of Hematology, The First Affiliated Hospital of Soochow University, Shizi Street 188, Suzhou, 215006 China; 2 Institute of Blood and Marrow Transplantation, Collaborative Innovation Center of Hematology, Soochow University, Suzhou, China; 3 Department of Emergency Medicine, Union Hospital, Tongji Medical College, Huazhong University of Science and Technology, Wuhan, China; 4 Department of Microbiology and Immunology, Life Sciences Institute, Immunology Translational Research ProgramYong Loo Lin School of MedicineImmunology ProgramNational University of Singapore, Singapore, Singapore

**Keywords:** IL-39, IL-12, Chronic graft-versus-host disease, Allogeneic hematopoietic stem cell transplantation

## Abstract

**Background:**

Chronic graft-versus-host disease (cGVHD) remains a major complication during the late phase of allogeneic hematopoietic stem cell transplantation (allo-HSCT). IL-39, a newly described pro-inflammatory cytokine belonging to the IL-12 family, plays a role in lupus development. Recently, IL-39 has been identified as a pathogenic factor in acute GVHD (aGVHD). However, the role of IL-39 in the pathogenesis of cGVHD remains unclear.

**Methods:**

We constructed a recombinant IL-39 plasmid and established scleroderma and lupus-like cGVHD models. Quantitative PCR and enzyme-linked immunosorbent assay (ELISA) were used to detect IL-39 expression in mice and patients post transplantation, respectively. Hydrodynamic gene transfer (HGT) was performed to achieve IL-39 overexpression in *vivo*. Multiparameter flow cytometry, western blotting, and assays in *vitro* were performed to investigate the effect of IL-39 on cGVHD.

**Results:**

The relative expression of IL-23p19 and EBi3 was significantly increased in the intestine of cGVHD mice on day 40 post allo-HSCT, and IL-39 levels were significantly elevated in the serum of patients following allo-HSCT. Overexpression of IL-39 significantly aggravated the severity of cGVHD. Increased IL-39 levels promoted T-cell activation and germinal center responses, and may exacerbate thymic damage. Consistently, blocking IL-39 markedly ameliorated immune dysregulation in the cGVHD mice. Furthermore, we found that IL-39 was produced by B cells, CD11b^+^ cells, and CD8^+^T cells after activation. Stimulation of IL-39 led to upregulation of the IL-39 receptor on CD4^+^T cells and further caused activation of the STAT1/STAT3 pathway, through which IL-39 may exert its pro-inflammatory effects.

**Conclusion:**

Our study reveals a critical role for IL-39 in cGVHD pathogenesis and indicates that IL-39 may serve as a potential therapeutic target for cGVHD prevention.

**Supplementary Information:**

The online version contains supplementary material available at 10.1186/s40164-022-00286-x.

## Background

Allo-HSCT is an effective method for the treatment of hematologic malignancies. However, cGVHD is a major complication of allo-HSCT, occurring in approximately 50% of patients [1–4]. Similar to aGVHD, donor T cells play a critical pathogenic role in initiating tissue injury and developing cGVHD [5]. Depleting mature T cells from stem cell grafts markedly reduces the incidence of cGVHD [6–9]. In addition to T cell activation, autoreactive B cells have also been found to play a pathogenic role in cGVHD. Under the action of high-level B cell activation factor (BAFF), more donor B cells differentiate into autoantibody-secreting subsets upon sustained stimulation of receptor antigens, resulting in pathological organ damage [10–12]. Therefore, exploring key molecules that cause immune dysregulation in T and B cells is expected to provide new mechanisms and targets for cGVHD prevention and treatment.

The IL-12 family consists of IL-12 (IL-12p35/IL-12p40), IL-23(IL-23p19/IL-12p40), IL-27 (IL-27p28/EBi3), IL-Y (IL-27p28/ IL-12p40), and IL-35 (IL-12p35/EBi3) [13, 14]. IL-12 and IL-23 play crucial roles in inducing differentiation of Th1 and Th17 cells, respectively [15]. IL-Y promotes cGVHD by activating pathogenic T and B cells. In contrast, IL-27 and IL-35 suppress inflammatory responses by promoting the expansion of regulatory B and T cell subsets [13, 15–17]. Wang et al. first described a new IL-12 member composed of IL-23p19 and an EBi3 heterodimer, IL-39 [18]. IL-39 was secreted by LPS-stimulated and GL7^+^ activated B cells and mediated inflammatory responses in lupus-like mice through activation of STAT1/STAT3 [18]. In addition, serum IL-39 levels were significantly increased and positively correlated with disease severity in patients with acute coronary syndrome (ACS) and neuromyelitis optica spectrum disorders [19, 20]. All of these studies indicate that IL-39 may play a pro-inflammatory role in immune regulation. Consistently, a recent study demonstrated that IL-39 was implicated in the pathogenesis of aGVHD, and one subunit of its receptor, IL-23Rα, played a role in promoting cGVHD development [21]. However, the role of IL-39 in cGVHD has not been well-defined.

In this study, we demonstrated that overexpression of IL-39 aggravated cGVHD, whereas blocking of IL-39 attenuated immune dysregulation in the cGVHD mice. IL-39 promoted T cell activation and increased the differentiation of germinal center (GC) B cells in mice following allo-HSCT. IL-39 augmented the secretion of pro-inflammatory cytokines, including IL-4 and TNF-α, and reduced the proportion of CD4^+^CD8^+^ double-positive cells in the thymus in cGVHD. IL-39 may be secreted by activated B cells, CD8^+^T cells, and CD11b^+^ cells, and further acted on CD4^+^T cells as well as CD8^+^T cells and stimulated the downstream STAT1/STAT3 signal pathway to facilitate the progression of cGVHD.

## Results

### IL-39 aggravates the development of cGVHD in mice

**Fig. 1:**
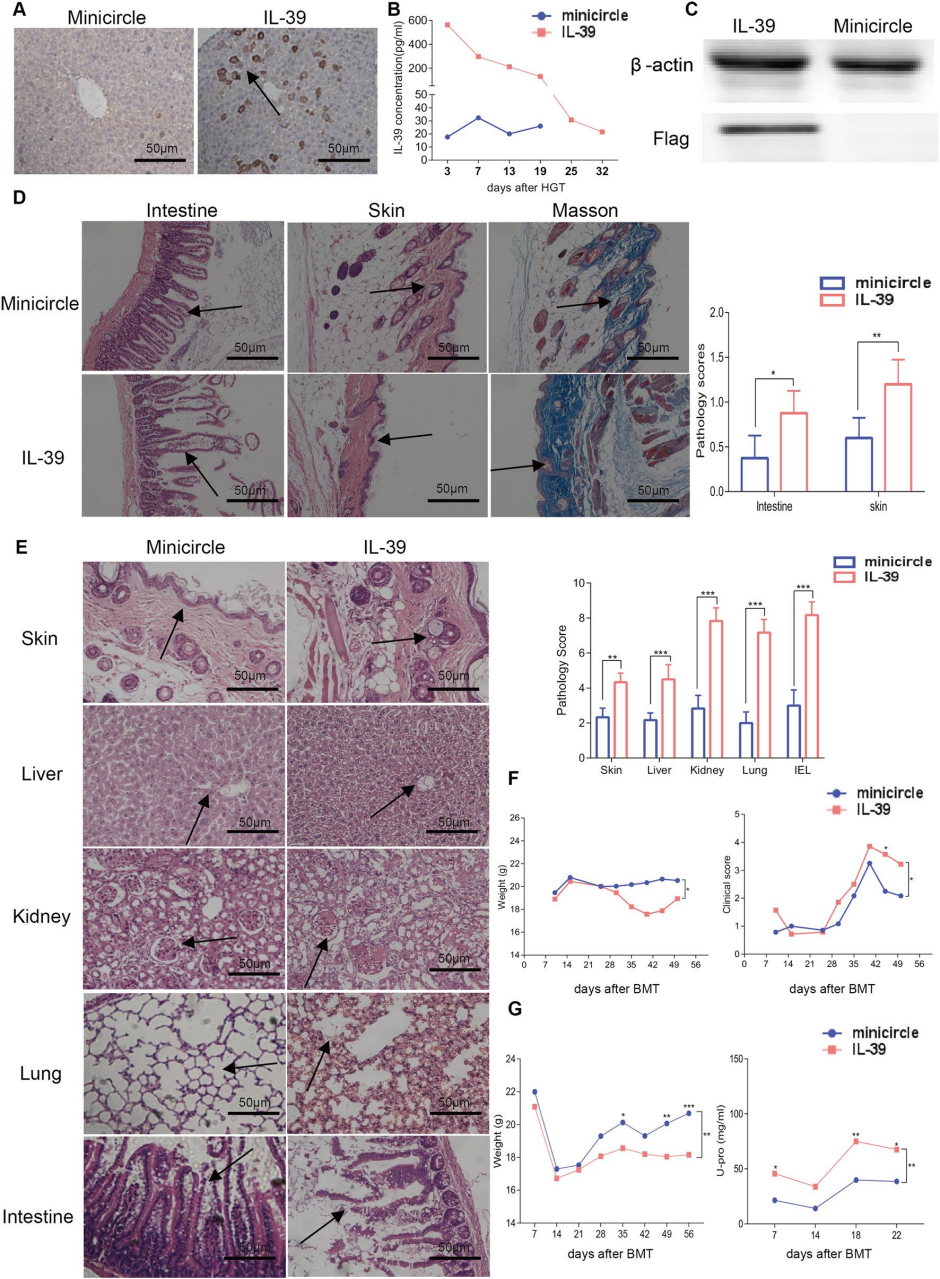
IL-39 aggravates the development of murine cGVHD. BALB/c mice were hydrodynamically injected with minicircles or IL-39 plasmids (n = 6 per group). Three days after HGT, irradiated BALB/c recipients were infused with 1 × 10^7^ bone marrow cells and 1 × 10^6^ splenocytes from C57BL/6 mice, to establish a scleroderma-like cGVHD model. IL-39 expression in the liver 7 days after HGT treatment was detected by immunohistochemistry (**A**) and western blotting (**C**). IL-39 levels in the serum were detected weekly using enzyme-linked immunosorbent assay (**B**). Representative histopathological images and pathology scores of the skin, intestine, and Masson’s trichrome staining are shown (**D**). Body weights and GVHD scores in scleroderma-like mice are shown (**F**). Three days after HGT, irradiated recipients were infused with 5 × 10^6^ bone marrow cells and 4 × 10^7^ CD25^−^ splenocytes from DBA/2 mice to establish a lupus-like cGVHD mouse model. Representative histopathological images and pathology scores of the skin, liver, intestine, kidney, and lung are shown (**E**). Body weights and urine protein levels in lupus-like mice are shown (**G**). The data are representative of at least three independent experiments. Values are presented as mean ± SD. *P < 0.05; **P < 0.01; ***P < 0.001

To study the involvement of IL-39 in cGVHD pathogenesis, we established a scleroderma-like cGVHD model and detected the expression of IL-39 in the lung, liver, small intestine, and spleen of recipients on days 30, 40, and 50 post transplantation. Compared with the control group (only donor bone marrow cells were transplanted), the relative expression of IL-23p19 and EBi3 was significantly increased in the intestine of cGVHD mice at 40 days post transplantation (*P* < 0.05), suggesting that the change in IL-39 expression may be related to cGVHD development (Additional file 1: Figure S1). To investigate the function of IL-39 in cGVHD induction, using a C57BL/6 to BALB/c cGVHD model, we injected plasmid containing Flag-tagged IL-39 gene or control plasmid into recipients 3 days before allo-HSCT using a hydrodynamic gene transfer method [13, 22]. Overexpression of IL-39 in the liver tissue was verified in mice that received the IL-39 plasmid (Fig. 1A). Serum IL-39 was also detectable up to approximately three weeks post-plasmid injection in these mice (Fig. 1B). Flag expression in the liver was confirmed by western blotting seven days after plasmid injection (Fig. 1C). To quantify the histopathology of cGVHD target organs, the skin and intestine of recipient mice were collected 56 days post transplantation. More severe tissue damage in the skin and small intestine was observed in recipients treated with IL-39 plasmid (Fig. 1D). Furthermore, we found increased collagen deposition in the skin of these mice, which was consistent with fibrosis as an important feature of cutaneous cGVHD (Fig. 1D). Although no survival difference was observed between IL-39 treated and control mice (Additional file 1: Figure S2A), treatment with IL-39 plasmid resulted in increased body weight loss and GVHD clinical scores in mice following allo-HSCT (Fig. 1F).

We further investigated the role of IL-39 in cGVHD pathogenesis using an established lupus-like cGVHD model [13]. Consistently, at 56 days after transplantation, we found more severe cGVHD in recipients treated with the IL-39 plasmid than in those treated with the minicircle control plasmid. The inflammatory response in the skin, liver, small intestine, lung, and kidney was increased, as reflected by the higher pathological scores in these organs from mice that received the IL-39 plasmid (Fig. 1E). Similar to the investigation in the scleroderma-like cGVHD model, we found no difference in survival between mice treated with IL-39 and control mice (Additional file 1: Figure S2B). The body weight of recipient mice treated with the IL-39 plasmid was markedly decreased compared to those treated with the minicircle from 5 weeks after allo-HSCT (Fig. 1G). Furthermore, higher proteinuria levels were found in recipients administered IL-39 plasmid than those in the minicircle group (Fig. 1G). Our data indicated that overexpression of IL-39 aggravates the severity of cGVHD.

### IL-39 promotes donor T-cell activation and reduces the proportion of CD4^+^CD8^+^ double-positive cells in the thymus during scleroderma-like cGVHD

**Fig. 2:**
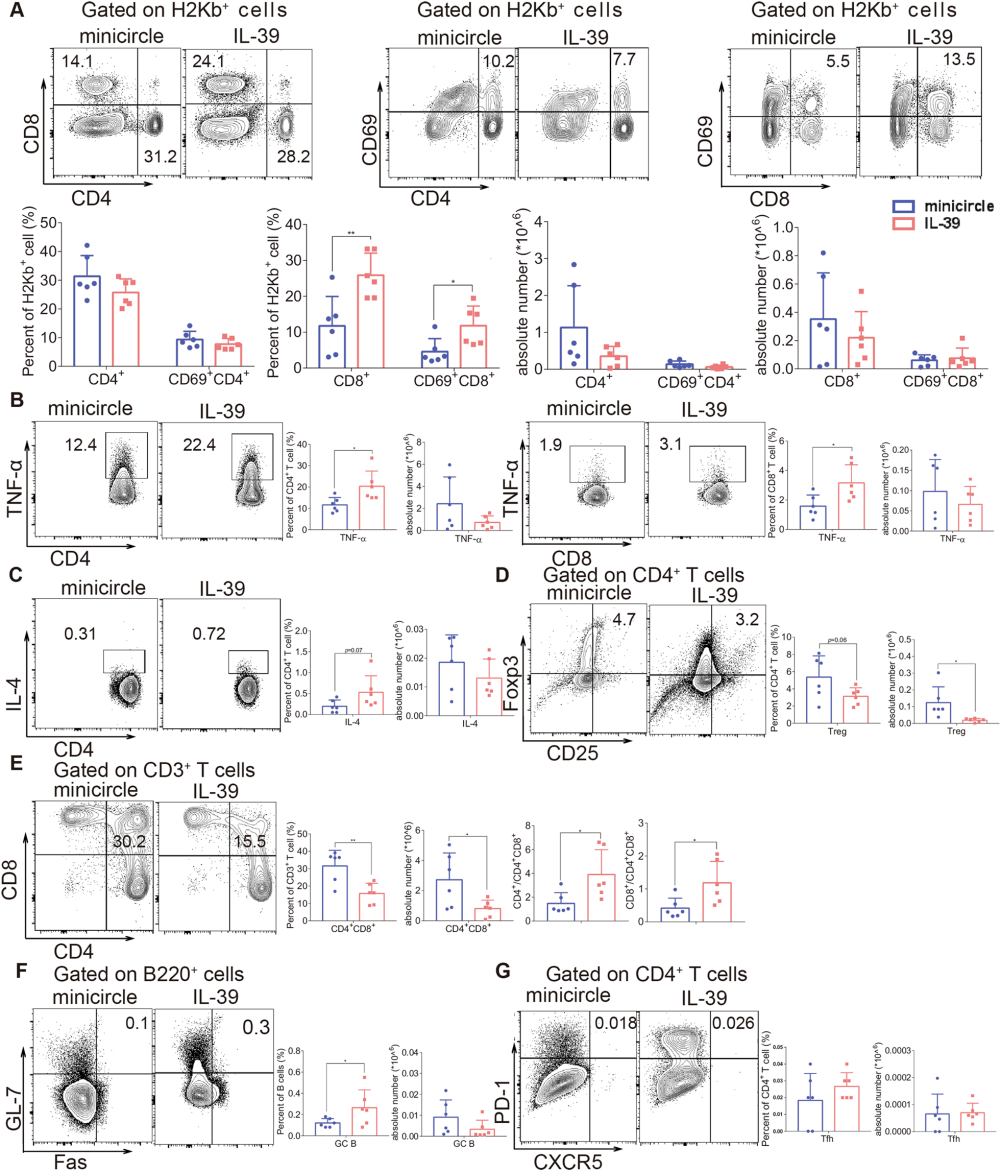
IL-39 promotes pro-inflammatory responses of T and B cells in the scleroderma-like cGVHD mice model. Irradiated BALB/c recipients were infused with 1 × 10^7^ bone marrow cells and 1 × 10^6^ splenocytes from the C57BL/6 mice. Splenocytes (n = 6 in each group) were collected and stained for FACS analysis eight weeks after transplantation. The percentages and numbers of CD4^+^, CD69^+^CD4^+^T, CD8^+^, and CD69^+^CD8^+^T cells in H2Kb^+^ cells from the spleens of recipients are shown (**A**). Lymphocytes were isolated from the spleens of recipients and treated with PMA, brefeldin A, and ionomycin for 4-6 h. The percentage and number of TNF-α-(**B**) and IL-4-(**C**) positive T cells in the spleens of recipients are shown. The percentage and number of Treg (CD4^+^CD25^+^Foxp3^+^) cells in the spleens of recipients are shown (**D**). The percentage and number of CD4^+^CD8^+^T cells and the CD4^+^/CD4^+^CD8^+^ and CD8^+^/CD4^+^CD8^+^ ratios in the thymus of recipients are shown (**E**). The percentage and number of Tfh (CD4^+^CXCR5^+^PD-1^+^) cells in the spleens of recipients are shown (**F**). The percentage and number of GC B (B220^+^GL-7^+^Fas^+^) cells in the spleens of recipients are shown (**G**). The data are representative of at least three independent experiments. Values are presented as mean ± SD. *P < 0.05; **P < 0.01

To further explore how IL-39 deteriorates the severity of cGVHD in recipients, we analyzed lymphocyte subsets in recipients with scleroderma-like cGVHD symptoms 8-week post transplantation. We found that the proportions of total splenic CD8^+^T cells and activated (CD69^+^) CD8^+^T cells in the recipients of the IL-39 group were significantly increased compared to those in the minicircle group (*P* < 0.01, *P* < 0.05). However, IL-39 had no significant effect on the proportion and number of total CD4^+^ T cells or activated CD4^+^T cells (Fig. 2A). We further detected intracellular cytokines and found that IL-39 overexpression upregulated the percentage of TNF-α-positive CD4^+^ and CD8^+^T cells in mice after allo-HSCT (Fig. 2B). IL-4 expression in CD4^+^T cells was slightly increased by the IL-39 plasmid treatment, but the difference was not statistically significant (Fig. 2C). These results suggest that IL-39 not only increases the percentage of CD8^+^T cells and promotes their activation, but also increases the proportion of TNF-α-producing T cells after allo-HSCT.

Treg cells maintain immune tolerance and prevent autoimmune and inflammatory diseases, including cGVHD [23–25]. We found that the percentage of Tregs was slightly reduced, whereas the absolute numbers were markedly decreased after IL-39 plasmid treatment (Fig. 2D). Previous studies have demonstrated that donor CD8^+^T cells impaired the negative selection process during T cell development in the thymus [9]. Based on our results that IL-39 can promote the activation of CD8^+^T cells, we speculated that IL-39 might aggravate thymic injury. In line with this hypothesis, the frequencies and absolute numbers of CD4^+^CD8^+^ thymocytes were significantly reduced in mice treated with the IL-39 plasmid compared to those in the control group (Fig. 2E). The ratio of single positive (SP) to double positive (DP) thymocytes was also markedly increased in mice administered the IL-39 plasmid (Fig. 2E). Our data indicated that injection of the IL-39 plasmid reduced Treg numbers and might aggravate thymic damage during scleroderma-like cGVHD development, suggesting its pathogenic role in the breakdown of immune tolerance (Fig. 2E).

### IL-39 promotes the differentiation of GC B cells in the spleens of scleroderma-like cGVHD recipients

To confirm whether IL-39 affects B-cell response during cGVHD, we harvested splenocytes from recipients with scleroderma-like cGVHD and examined the expression of surface markers of B lymphocytes, including B220, Fas (CD95), GL-7, and CD138 in recipient mice on day 56 post allo-HSCT. Analysis of B cell subsets indicated that the ratio, but not the absolute number of GC B cells (GL7^+^CD95^+^), was significantly increased in recipients with IL-39 plasmid administration (Fig. 2F). T follicular helper (Tfh) cells also play a vital role in the pathogenesis of cGVHD by supporting B-cell activation and promoting GC B-cell differentiation [5, 12]. However, the percentage and absolute number of Tfh (CD4^+^CXCR5^+^PD-1^+^) cells were comparable between the two groups (Fig. 2G). These data suggest that IL-39 promotes germinal center responses by enhancing GC B cell differentiation during cGVHD development.

### IL-39 increases T- and B-cell responses during the development of lupus-like cGVHD

**Fig. 3:**
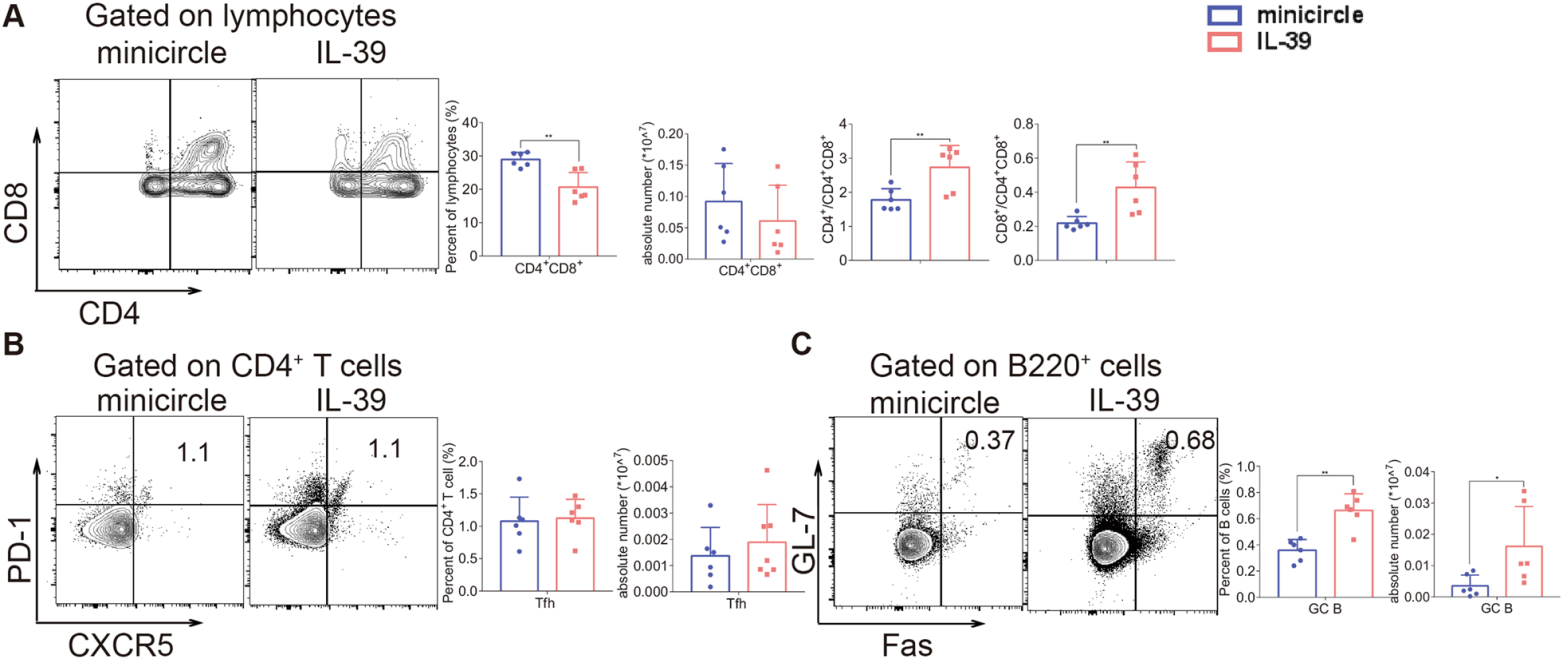
IL-39 promotes the pro-inflammatory responses of T and B cells in the lupus-like cGVHD mice model. Irradiated BALB/c recipients were infused with 5 × 10^6^ bone marrow cells and 4 × 10^7^ CD25^−^ splenocytes from DBA/2 mice. Splenocytes (n = 6 per group) were collected and stained for FACS analysis 8 weeks post transplantation. The percentage and number of CD4^+^CD8^+^T cells and the CD4^+^/CD4^+^CD8^+^ and CD8^+^/CD4^+^CD8^+^ ratios in the thymuses of recipient mice are shown (**A**). The percentage and number of Tfh (CD4^+^CXCR5^+^PD-1^+^) cells in the spleens of recipients are shown (**B**). The percentage and number of GC B (B220^+^GL-7^+^Fas^+^) cells in the spleens of recipients are shown (**C**). The data are representative of at least three independent experiments. Values are presented as mean ± SD. *P < 0.05; **P < 0.01

To exclude the possibility of model specificity, we established a lupus-like cGVHD model, and explored the mechanisms underlying IL-39 pathogenicity in cGVHD. As we could not distinguish between donor and recipient cells in this MHC-matched model, we analyzed whole T and B cells in the recipient spleens. We found that the percentages of activated CD4^+^T cells and CD8^+^T cells were increased in the spleens of recipients who received the IL-39 plasmid compared to those receiving the minicircle (Additional file 1: Fig. S3A). Consistent with the findings in the scleroderma-like cGVHD model, we found that the frequencies of TNF-ɑ-and IL-4-expressing CD4^+^T cells were significantly increased in the spleens of lupus-like recipients with IL-39 plasmid treatment (Additional file 1: Fig. S3B and S3C). Although not statistically significant, there was a small increase in the proportion of TNF-α-producing CD8^+^T cells (Additional file 1: Fig. S3B). However, IL-39 plasmid treatment in this cGVHD model did not affect the percentage or absolute number of Treg cells (Additional file 1: Fig. S3D). We found that the frequency of CD4^+^CD8^+^ thymocytes was significantly decreased, while the ratios of CD4^+^/DP and CD8^+^/DP were significantly elevated in IL-39 plasmid-treated recipients (Fig. 3A), indicating that IL-39 could exacerbate thymic damage in both lupus-like and scleroderma-like cGVHD models. In addition, we did not observe a significant difference in the percentage or absolute number of Tfh cells in the lupus-like model (Fig. 3B), whereas the frequency and number of splenic GC B cells increased in IL-39-overexpressing mice (Fig. 3C). Taken together, our data indicate that IL-39 infusion enhances T cell activation and GC B cell differentiation, while reducing the proportion of CD4^+^CD8^+^ double-positive cells in the thymus, thereby aggravating lupus-like cGVHD.

### Blocking IL-39 ameliorates the immune dysregulation during scleroderma-like cGVHD

**Fig. 4:**
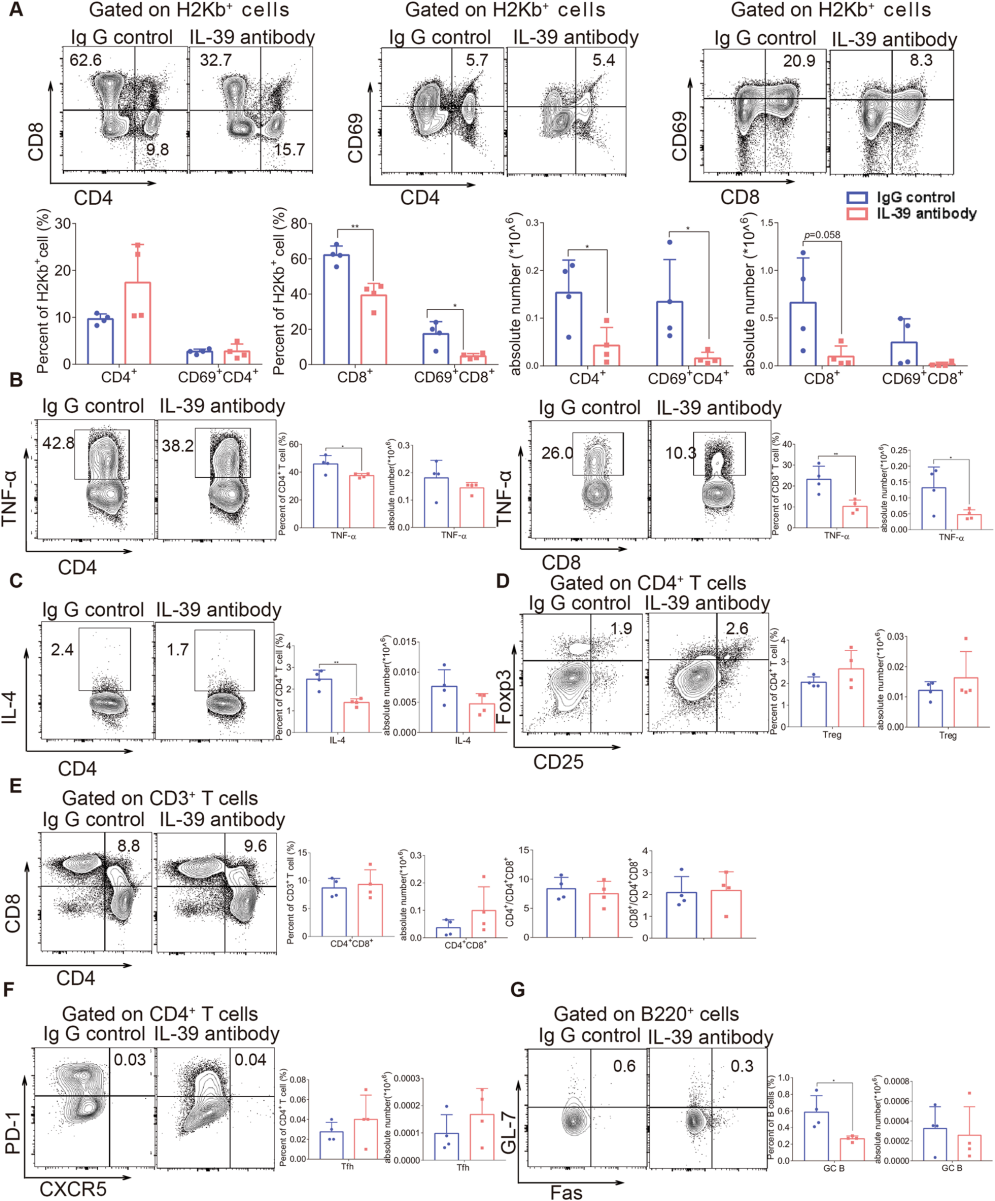
Anti-IL-39 antibody ameliorates immune dysregulation in scleroderma-like cGVHD. Irradiated BALB/c recipients were infused with 1 × 10^7^ bone marrow cells and 1 × 10^6^ splenocytes from the C57BL/6 mice. Fourteen days after transplantation, each mouse in the antibody group received 100 μl (100 μg) of IL-39 antibody, while each mouse in the control group received 100 μl (100 μg) of isotype control antibody via intraperitoneal injection twice a week for 6 weeks. Splenocytes (n = 4 in each group) were collected and stained for FACS analysis eight weeks after transplantation. The percentages and numbers of CD4^+^, CD69^+^CD4^+^T, CD8^+^, and CD69^+^CD8^+^T cells in H2Kb^+^ cells from the spleens of recipients are shown (**A**). Lymphocytes were isolated from the spleen and treated with PMA, brefeldin A, and ionomycin for 4-6 h. The percentage and number of TNF-α-(**B**) and IL-4-(**C**) positive T cells in the spleens of recipients are shown. The percentage and number of Treg (CD4^+^CD25^+^Foxp3^+^) cells in the spleens of recipients are shown (**D**). The percentage and number of CD4^+^CD8^+^T cells and the CD4^+^/CD4^+^CD8^+^ and CD8^+^/CD4^+^CD8^+^ ratios in the thymuses of recipients are shown (**E**). The percentage and number of Tfh (CD4^+^CXCR5^+^PD-1^+^) cells in the spleens of recipients are shown (**F**). The percentage and number of GC B (B220^+^GL-7^+^Fas^+^) cells in the spleens of recipients are shown (**G**). The data are representative of at least three independent experiments. Values are presented as mean ± SD. *P < 0.05; **P < 0.01

Given that increased IL-39 levels exacerbated pro-inflammatory immune responses during cGVHD, we investigated whether blocking IL-39 could ameliorate immune dysregulation in the scleroderma-like cGVHD model without artificial IL-39 overexpression. We first examined the effectiveness of the anti-IL-39 antibody in *vitro* and found that anti-IL-39 antibody suppressed the secretion of pro-inflammatory cytokines in T cells (Additional file 1: Figure S4). We next used a scleroderma-like cGVHD model to investigate the in vivo effect of anti-IL-39 on pro-inflammatory immune responses. We observed that no mice died in either group during the entire experimental period, except for one that died before antibody infusion (Additional file 1: Figure S5A). Body weight was significantly higher in IL-39 antibody-treated mice than in control mice (*P* < 0.01), and there was also a significant decrease in the GVHD clinical score in IL-39 antibody-treated mice compared with control mice on day 42 (Additional file 1: Figure S5B, *P* < 0.05). We found that blocking IL-39 significantly decreased the frequencies of CD8^+^T and CD69^+^CD8^+^T cells and reduced the numbers of CD4^+^T and CD69^+^CD4^+^T cells in recipient spleens compared to those treated with the isotype control antibody (Fig. 4A). There was a slight decrease in the absolute number of CD8^+^T cells in the spleens of anti-IL-39 antibody-treated mice (*P* = 0.058) (Fig. 4A). Furthermore, blocking IL-39 significantly attenuated the percentage of TNF-α-or IL-4-producing CD4^+^T cells and reduced the proportion and absolute number of TNF-α-producing CD8^+^T cells (Fig. 4B, C). Accordingly, the immune regulatory function of IL-39 inhibition can be investigated in target tissues. TNF-α expression in CD8^+^T cells and IL-4 expression in CD4^+^T cells were downregulated in the intestine (Additional file 1: Figure S6A). The numbers of CD8^+^T cells and CD69^+^CD4^+^T cells decreased in the liver (Additional file 1: Figure S6B, *P* < 0.05). The number and percentage of CD8^+^T cells decreased in the lungs (Additional file 1: Figure S6C, *P* < 0.05). There was no difference in the proportion or number of Tregs between the anti-IL-39 antibody and isotype control antibody treatment groups (Fig. 4D). Anti-IL-39 treatment did not affect the frequency of CD4^+^CD8^+^ thymocytes or the SP/DP ratio (Fig. 4E). Although the percentage and number of Tfh cells were not affected by the IL-39 blockade (Fig. 4F), the proportion of splenic GC B cells was significantly reduced in anti-IL-39-treated mice (Fig. 4G). Taken together, these data suggest that the administration of anti-IL-39 antibodies might alleviate immune dysregulation during cGVHD by controlling T cell expansion and activation, suppressing pro-inflammatory cytokine production of donor T cells, and attenuating GC responses.

### IL-39 is positively correlated with the severity of cGVHD in patients

**Fig. 5:**
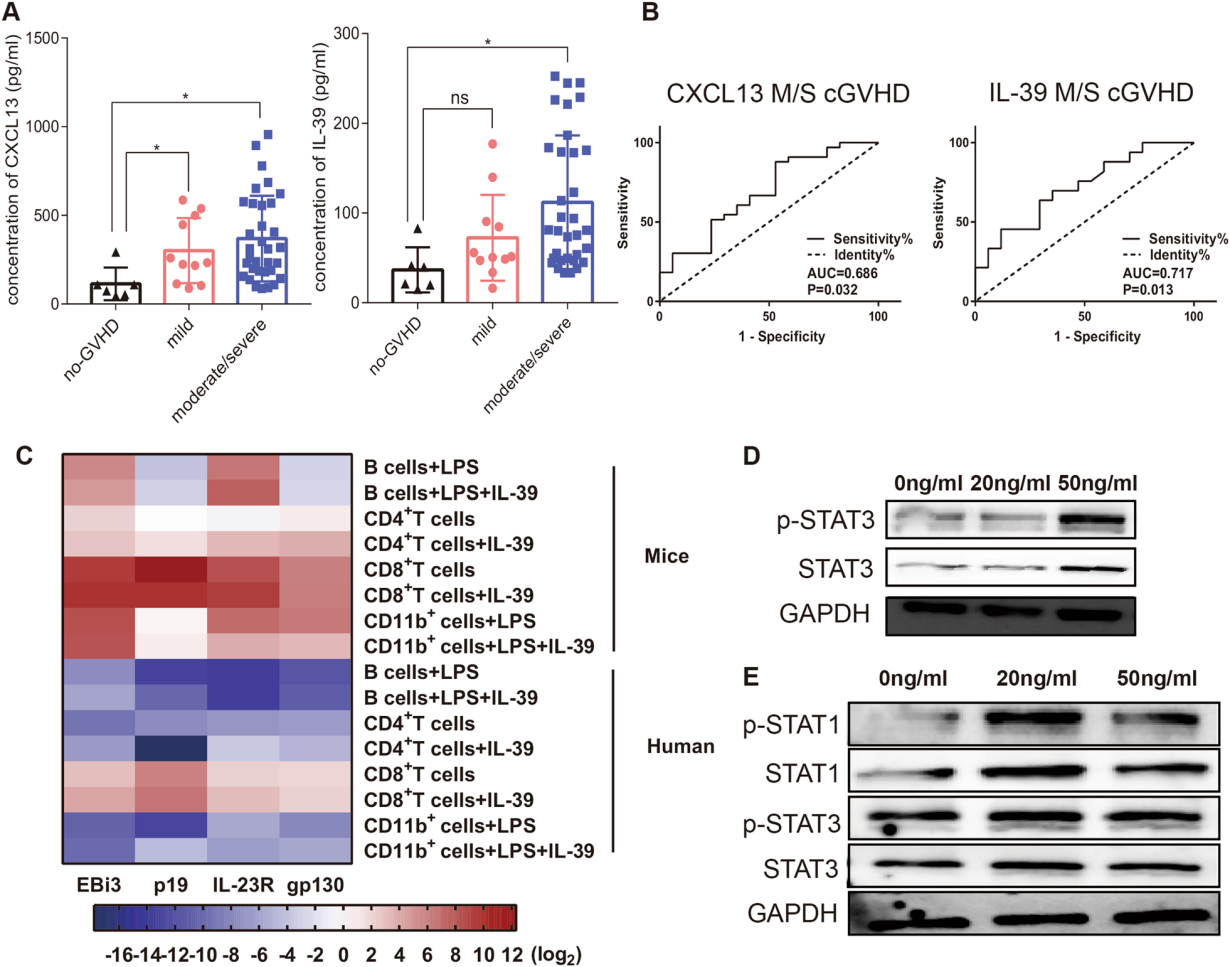
Serum IL-39 levels were significantly elevated in patients with cGVHD. CXCL13 and IL-39 levels in serum were detected in non-GVHD, mild, and moderate/severe cGVHD patients (**A**). The prognostic value of CXCL13 and IL-39 in no-cGVHD and mild and moderate/severe cGVHD patients was evaluated using ROC curves (**B**). CD4^+^T cells, CD8^+^T cells, B cells, and CD11b^+^ cells were sorted from the splenocytes of C57BL/6 mice or PBMCs from healthy donors using a Cell Isolation Kit according to the manufacturer’s protocol. For CD4^+^ and CD8^+^T cell activation, plates were coated with 2 mg/ml anti-CD3 and 0.4 mg/ml anti-CD28 Abs overnight. Next, 2 × 10^5^ T cells were cultured with recombinant mouse or human IL-39 proteins (20 ng/ml) for 72 h. For B cell and CD11b^+^ cell activation, 2 × 10^5^ B cells and CD11b^+^ cells were cultured with LPS (5 ng/ml) and recombinant mouse or human IL-39 proteins (20 ng/ml) for 72 h. The relative expression of IL-39 and IL-39R genes was determined by real-time PCR in CD4^+^T cells, CD8^+^T cells, B cells, and CD11b^+^ cells from the splenocytes of C57BL/6 mice and PBMCs from healthy donors (**C**). In the following experiments, plates were coated with 2 mg/ml anti-CD3 and 0.4 mg/ml anti-CD28 Abs overnight. T cells (2 × 10^5^ T cells were cultured with various concentrations of recombinant mouse or human IL-39 proteins for 72 h. Phosphorylation of STAT1/STAT3 detected by western blotting in sorted primary T cells from mice or healthy donors is shown (**D**, **E**). The data are representative of at least three independent experiments. Values are presented as mean ± SEM. *P < 0.05; ***P < 0.001

To explore the effect of IL-39 on clinical cGVHD, we performed a retrospective analysis of patients who developed cGVHD after allo-HSCT and those who did not. According to the NIH consensus criteria, we grouped the patients into no cGVHD, mild cGVHD, and moderate/severe (M/S) cGVHD groups. In addition, CXCL13 has been previously suggested as an essential indicator of the occurrence and development of cGVHD [26, 27]. To further describe the disease status of patients, we detected serum CXCL13 levels, and found that the average concentration of CXCL13 was 113.5 pg/ml, 301.0 pg/ml, and 368.6 pg/ml in patients with no cGVHD, mild cGVHD, and moderate/severe (M/S) cGVHD group, respectively (Additional file 1: Table S4). We then measured IL-39 levels in the corresponding patient groups. We found that IL-39 concentrations were higher in patients with cGVHD than in those without (Fig. 5A). Furthermore, IL-39 levels showed an increasing trend in patients with M/S cGVHD compared to those with mild cGVHD (Fig. 5A). When comparing patients with or without M/S cGVHD, the AUC of CXCL13 and IL-39 were 0.686 (95% CI 0.528–0.844, *P* = 0.032) and 0.717 (95% CI 0.569–0.865, *P* = 0.013), respectively. The CXCL13 cut-off value was 125.78 pg/ml from the ROC curve, and the sensitivity and specificity were 87.9% and 47.1%, respectively. The IL-39 cut-off value was 52.44 pg/ml, the sensitivity and specificity were 69.7% and 64.7%, respectively (Fig. 5B). Therefore, our data indicate a positive correlation between IL-39 levels and cGVHD development, suggesting that high IL-39 levels may be a predictor of M/S cGVHD development in patients post allo-HSCT.

### IL-39 affects the expression of itself and its receptor on immune cells and exerts a pro-inflammatory response in T cells via STAT1/STAT3 signaling pathway

We further investigated potential downstream pathways in immune cells that respond to IL-39 stimulation. Currently, there are no specific antibodies against the IL-39 receptor. Therefore, we examined IL-39 or IL-39 receptor-related gene expression in sorted CD19^+^B cells, CD4^+^T cells, CD8^+^T cells, and CD11b^+^cells in mice and humans. We examined IL-39 heterodimers, including IL-23p19 and EBi3, as well as IL-39 receptor heterodimers, including IL-23R and gp130. Surprisingly, we found that although LPS (5 ng/ml) activated B cells, CD11b^+^ cells, and anti-CD3/CD28 stimulated CD4^+^T cells produced moderate amounts of genes encoding heterodimers of IL-39 and its receptor, both mouse- and human-derived activated CD8^+^T cells expressed high levels of EBi3, IL-23p19, IL-23R, and gp130 (Fig. 5C). Interestingly, the addition of IL-39 only slightly affected the expression of genes encoding IL-39 and its receptor in CD8^+^T cells, while significantly promoting the expression of IL-23R and gp130 in CD4^+^T cells (Fig. 5C). Moreover, IL-39 administration promoted the expression of IL-23p19 in B cells as well as the expression of both EBi3 and IL-23p19 in CD11b^+^ cells (Fig. 5C). Taken together, these findings suggested that activated CD8^+^T cells might produce massive amounts of IL-39 and express high levels of IL-39 receptor, regardless of whether they were IL-39 treated or not, whereas IL-39 administration mainly increased the expression of IL-39 receptor on CD4^+^T cells and promoted the production of IL-39 on B cells and CD11b^+^cells.

We next investigated whether IL-39 exerted its pro-inflammatory effects on T cells by activating the downstream STAT pathways. We found that the phosphorylation of STAT3 was significantly increased in sorted murine primary T cells co-cultured with 50 ng/ml mouse recombinant IL-39 protein (Fig. 5D). However, we did not observe the activation of the STAT1 pathway (Additional file 1: Figure S7). We also isolated T cells from peripheral blood mononuclear cells (PBMCs) of healthy donors and co-cultured them with recombinant human IL-39 protein. Interestingly, we found that rIL-39 significantly augmented the phosphorylation of STAT1 rather than STAT3 in human T cells (Fig. 5E). These data suggest that IL-39 may mediate inflammatory responses through activation of STAT3 in murine primary T cells and STAT1 in human T cells.

## Discussion

In the current study, we demonstrated that IL-39 expression was increased in murine and human cGVHD, and that overexpression of IL-39 aggravated the development of cGVHD in two different murine cGVHD models. IL-39 played a pro-inflammatory role in regulating the function of multiple immune cells involved in the pathogenesis of cGVHD, including TNF-α-producing T cells, Th2 cells, and B cells. Consistently, application of an anti-IL-39 antibody effectively reduced immune dysregulation in a classic scleroderma-cGVHD murine model. In vitro experiments indicated that activated B cells, CD8^+^T cells, and CD11b^+^ cells can produce IL-39, which might promote the expression of IL-39 receptors on CD4^+^T cells. IL-39 may promote STAT3 phosphorylation in murine T cells and activate the STAT1 pathway in human T cells via interaction with the IL-39 receptor on T cells. Taken together, our results demonstrate that IL-39 plays an essential role in the pathophysiology of cGVHD by promoting the pro-inflammatory responses of T and B cells.

Previous studies have suggested that T cells undergo both positive and negative selection within the thymus, resulting in the elimination of self-reactive cells, which play a pathogenic role in the induction of cGVHD [28]. The thymus can be damaged by prior chemotherapy, conditioning regimens, and aGVHD, and such thymic injury results in a loss of thymic negative selection [8]. Our results showed that the proportion of CD4^+^CD8^+^T cells in the thymus was significantly decreased, whereas the SP/DP ratio was increased in recipients treated with IL-39. However, the frequency of CD4^+^CD8^+^ cells was not obviously elevated in IL-39-blockade recipients, indicating that IL-39 could aggravate immune dysregulation by modulating T-cell activation in addition to thymic damage. Additionally, the effects of infusion or inhibition of IL-39 on thymus damage still need to be further verified by performing thymus histology and analysis of thymic epithelial and fibroblastic cell populations.

The pathophysiology of cGVHD mostly revolves around the polarization of CD4^+^T cells to the Th2 cytokine phenotype, leading to the emergence of autoreactive B cells and the production of autoreactive antibodies [29]. In clinical allo-HSCT, TNF-α levels, either in the serum or blood leukocytes, are correlated with the severity of GVHD [30]. It has been reported that TNF-α may be a major mediator in the pathophysiology of the gastrointestinal tract and skin GVHD [31]. We demonstrated that IL-39 could play a pathogenic immune role by increasing the proportion of Th2 cells and secretion of TNF-α in CD4^+^ and CD8^+^T cells in scleroderma-like and lupus-like cGVHD models. Moreover, the expression of pro-inflammatory cytokines in T cells decreased with IL-39 blockade, consistent with the role of anti-IL-39 polyclonal antibodies in mitigating autoimmune symptoms in lupus-like mice [32]. Although we did not find obvious changes in Th1, Th17, Tc1, and Tc17 cytokine expressions in the spleens of cGVHD mice upon IL-39 treatment (Additional file 1: Figure S8), it is unclear whether IL-39 could affect other functions of T and B cells. Treg cells have been shown to be critically involved in immune tolerance induction to allo-antigens [23]. Evidence from clinical reports has demonstrated that infusion of donor Treg cells can reduce GVHD severity [33, 34]. We observed that the frequency of Treg cells was significantly decreased in IL-39-overexpressing mice during scleroderma-like cGVHD, while IL-39 treatment showed no effect on the Treg cell proportion in lupus-like cGVHD, indicating that the effect of IL-39 on Treg cells was likely to be variable in different cGVHD models.

Donor B cells play critical roles in the progression of cGVHD by acting as antigen-presenting cells (APCs) and augmenting the clonal expansion and survival of pathogenic CD4^+^T cells [35]. In our study, the level of GC B cells increased significantly, while the frequency of total B cells was comparable in mice administered IL-39, suggesting that IL-39 could aggravate the development of cGVHD by enhancing the differentiation of GC B cells in the mouse model. Nevertheless, given the lack of histological examination of the spleens or lymph nodes in our study, we cannot conclude that IL-39 treatment affects the formation of GCs. The interaction between Tfh cells and B cells occurs at the extrafollicular T-B border and follicular GCs, and promotes the differentiation of naive B cells, generation of GCs, and the production of class-switched antibodies [12, 36, 37]. However, we did not observe a significant increase in activated Tfh cells in the IL-39 group, suggesting that IL-39 may promote the differentiation of B cells through the assistance of other types of helper T cells, such as Th2 cells, during cGVHD.

Previous studies have shown that IL-39 is secreted by LPS-stimulated B cells and GL7^+^ activated B cells in lupus-like mice [18]. Consistently, we found that IL-39 administration significantly increased the expression levels of genes encoding IL-23p19 and EBi3 in LPS-activated B cells and CD11b^+^ cells isolated from murine splenocytes and human PBMCs. We also found that the administration of IL-39 mainly upregulated the expression of IL-39 receptors on activated CD4^+^T cells instead of B cells, CD8^+^T cells or CD11b^+^ cells in mice and humans. Unexpectedly, both mice- and human-derived activated CD8^+^T cells expressed relatively high levels of genes encoding IL-39 and its receptor, whereas IL-39 administration did not affect their expression in CD8^+^T cells. Nevertheless, we were unable to determine which cell type is mainly responsible for IL-39 production in vivo and which IL-39 secreting cells are critical for cGVHD development. Murine cGVHD models with cells (CD4^+^T cells, CD8^+^T cells, B cells, or CD11b^+^ cells) lacking IL-39 expression will be required to fully address this point. Interestingly, our data indicated that both overexpression and inhibition of IL-39 were associated with changes in the absolute numbers and activation of CD8^+^T cells rather than those in CD4^+^T cells. Considering the critical role of CD4^+^T cells in inducing cGVHD, we also investigated the effects of IL-39 overexpression on CD4^+^ and CD8^+^T cells at earlier time points (4 weeks post transplantation). However, we did not observe obvious differences in the activation, cytokine production, and the percentage and absolute number of CD4^+^ and CD8^+^T cells between IL-39 treated mice and control mice at this time point (Additional file 1: Figure S9). Donor CD8^+^T cells have been shown to be potent inducers in the C57BL/6 → BALB/c cGVHD model by exacerbating thymic damage [9]. Accordingly, we also investigated the significantly reduced frequencies and absolute numbers of CD4^+^CD8^+^ thymocytes in cGVHD mice treated with the IL-39 plasmid, suggesting that CD8^+^T cells are likely to play a major role in the IL-39-induced pathogenic effects on cGVHD. However, changes in IL-4 and TNF-α expression in CD4^+^T cells may be equally important for the pathogenic role of IL-39. Further experiments are needed to confirm this hypothesis. Taken together, IL-39 might be produced not only by activated B cells and CD11b^+^ cells but also by activated CD8^+^T cells, which subsequently leads to enhanced CD4^+^T cell responses to IL-39 by promoting IL-39 receptor expression on CD4^+^T cells. Activated CD4^+^T cells may provide positive feedback on the activation of B and CD8^+^T cells, which eventually aggravates cGVHD. However, it should be noted that we did not observe the effects of IL-39 overexpression in normal mice; therefore, we cannot rule out the possibility that inflammation induced by IL-39 treatment itself may also contribute to cGVHD development. Further experiments are required to validate this hypothesis.

Previous studies have shown that the EBi3-related cytokines IL-27 and IL-35 mediate their effects by activating the STAT pathways in B cells [38, 39]. Indeed, Wang et al. showed that IL-39 could play an important role in the pathophysiology of autoimmune diseases through the activation of the STAT1/STAT3 pathways [18]. Interestingly, we found elevated phosphorylation of STAT3 levels instead of STAT1 in sorted murine CD3^+^T cells, and elevated phosphorylation of STAT1 levels instead of STAT3 in human primary T cells sorted from PBMCs, indicating that, during cGVHD, IL-39 might exert pro-inflammatory effects by respectively activating STAT1 and STAT3 in humans and mice through interactions with IL-23R/gp130.

Bastian et al. reported that IL-23Rα played a vital role in donor T cell pathogenicity during cGVHD but did not find significantly increased serum IL-39 concentrations in cGVHD mice [21]. In contrast to their results, using the same cGVHD model, we observed that IL-39 levels were higher in the intestine of cGVHD mice 40 days after allo-HSCT compared to the control group (Additional file 1: Figure S1). Such inconsistent results may be due to the differential expression or distribution of IL-39 in organs and peripheral blood. Another possible explanation for the discrepancy could be related to the different dosages of splenocytes transferred (0.35 × 10^6^ vs. 1 × 10^6^), which might cause different severities of cGVHD. We found that IL-39 concentrations were higher in patients with cGVHD than in those without cGVHD (Fig. 5A). Moreover, IL-39 levels showed an increasing trend in patients with M/S cGVHD compared to those with mild cGVHD (Fig. 5A), suggesting that different cGVHD severities might lead to different levels of IL-39 expression and its subsequent functions.

Despite its high expression in the intestine of cGVHD mice, we found that the expression of IL-23p19 and EBi3 varied in different organs at different time points (Additional file 1: Figure S1). Due to pre-transplantation conditioning, severe injury may occur earlier in the intestine than in other organs during cGVHD development. Therefore, severe injury and cell death may occur earlier in the intestine than in other organs. Indeed, our histological examination on day 56 post-transplantation revealed severe damage in the intestine after IL-39 treatment, whereas the liver and lung only exhibited large immune cell infiltration rather than tissue damage (Fig. 1E). We speculated that on day 50 post-transplantation, excess cell death might lead to reduced cell numbers in the intestine of cGVHD mice, therefore resulting in decreased IL-39 expression. In contrast, abundant immune cell infiltration in the liver and lungs of cGVHD mice at this time point led to increased cell numbers and may have resulted in upregulation of IL-39 expression. Meanwhile, increased immune cell migration from the spleen to the liver and lung would decreased the absolute number of splenocytes in cGVHD mice, which might subsequently reduce IL-39 expression in the spleen.

Our results demonstrated that IL-39 levels were upregulated in patients with cGVHD and closely related to the progression of clinical cGVHD. ROC curve analysis also indicated that IL-39 was a risk factor for M/S cGVHD development (Fig. 5B). Therefore, we speculated that the upregulation of serum IL-39 might be a potential prognostic and diagnostic biomarker for patients with cGVHD. Nevertheless, multicenter studies with larger sample size are required for verification. Moreover, our study has some limitations. The primary cellular source of increased IL-39 expression is unclear, and the specific cellular and molecular mechanisms underlying the role of IL-39 in cGVHD are not well defined. Given the lack of long-term survival outcomes and the results of histological damage of target tissues upon IL-39 inhibition, we are not able to exclusively conclude that targeting IL-39 could alleviate cGVHD development. Additionally, although no increase in IFN-γ^+^ CD8^+^ T cells was found after IL-39 blockade, we did not use a combined cGVHD/graft versus leukemia (GVL) model to directly demonstrate the effect of IL-39 inhibition on GVL activity. Therefore, whether IL-39 represents a potential target for the separation of cGVHD and GVL responses after allo-HSCT remains unclear. Further investigations are warranted to pave the way for targeting IL-39 in cGVHD treatment after allo-HSCT.

## Conclusion

All the above results, both in mice and human samples, confirmed that IL-39 levels were strongly associated with cGVHD development. Here, we provide direct evidence for IL-39 pathogenic role in aggravating cGVHD by activating T and B cells and promoting pro-inflammatory cytokine secretion. Meanwhile, IL-39 inhibition alleviated immune dysregulation in cGVHD mice. IL-39 likely exerts its pro-inflammatory effects by activating STAT3 phosphorylation in murine T cells and the STAT1 pathway in human T cells. Our study suggests that targeting IL-39 may be beneficial during cGVHD treatment.

## Material and methods

### Plasmid construction

cDNA encoding mouse IL-23p19 and EBi3 was amplified by PCR from the total RNA extracted from LPS-treated splenocytes of C57BL/6 mice. The primer sequences used were as follows: EBi3F: 5'- GGTACCGCTCTCGTGGCTCTAAGCC -3'; EBi3R: 5'- GGATCCGGGCTTATGGGGTGCACTT -3'; IL-23p19F: 5'- ATGCTGGATTGCAGAGCAGTAA -3'; IL-23p19R: 5'- AGCTGTTGGCACTAAGGGCTCAGT -3'. 45 bases of coding (gly4ser) junctions were introduced between EBi3 and IL-23p19 using overlapping PCR techniques. The IL-39 expression construct was inserted between the EcoR I and Xbal I sites into minicircle (MC) plasmid (pMC.EF1; SBI, Palo Alto, CA, USA).

### Mice

6–8-week-old female C57BL/6 (H-2^b^) and BALB/c (H-2^d^) mice were purchased from SLAC Animal Laboratory (Shanghai, China). 8–10-week-old female DBA/2 mice (H-2^d^) were purchased from Charles River Laboratories (Beijing, China). The experimental animals were kept under specific pathogen-free (SPF) conditions.

### Establishment of murine models of cGVHD

One week before setting up the experiments, recipient mice were fed gentamicin aqueous solution to prevent intestinal infections. For HGT, recipient mice (BALB/c) were injected i.v. with 120 μg of recombinant plasmid in a total of 2 ml PBS within 5 s at 3 days before transplantation. Recipient BALB/c mice were given total body irradiation (TBI) at 650 cGy using a RAD 320 X-ray Irradiator 6–8 h prior to transplantation. To establish a scleroderma-like cGVHD model, recipients were infused with 1 × 10^7^ bone marrow cells and 1 × 10^6^ splenocytes from the C57BL/6 mice. To establish a lupus-like cGVHD model, irradiated recipients (BALB/c mice) were infused with 5 × 10^6^ bone marrow cells and 4 × 10^7^ CD25^−^ splenocytes from DBA/2 mice. To investigate the preventable effect of anti-IL-39 on cGVHD, 14 days after transplantation, mice received 100 μl (100 μg) of anti-IL-39 antibody (DETAIBIO, China) or 100 μl (100 μg) of non-specific IgG (BioXcell, BE0095) via intraperitoneal injection twice a week for 6 weeks.

### Histology

Representative samples of the lung, liver, small intestine, skin, and kidney were obtained from transplanted recipients, fixed in 4% formalin, and stained with H&E. The pathology score of the target organs was based on a previous scoring system [40, 41].

### Flow cytometry

The antibodies used for flow cytometry are listed in Additional file 1: Table S3. For intracellular staining, cells were treated with brefeldin A (BFA, 10 μg/ml) (Biolegend, San Diego, CA), phorbol-12-myristate-13-acetate (PMA, 50 ng/ml) (Beyotime Biotechnology, China), and ionomycin (500 ng/ml) (Beyotime Biotechnology, China) in a 37 °C cell culture incubator containing 5% CO_2_. The cells were then fixed and permeabilized with FACS Permeabilizing Solution (BD Biosciences, San Diego, CA, USA). Foxp3 staining kit was purchased from eBioscience (San Diego, CA, USA). Data were acquired using FACS NovoCyte (ACEA Biosciences, San Diego, CA, USA) and analyzed using FlowJo software (FlowJo, Ashland, OR, USA).

### Patients and sample preparation

Fifty patients who underwent hematopoietic stem cell transplantation at the First Affiliated Hospital of Soochow University between April 2015 and August 2017 were enrolled. We grouped the patients into no cGVHD, mild cGVHD, and moderate/severe (M/S) cGVHD groups according to the NIH consensus criteria [42]. The characteristics of the patients are shown in Additional file 1: Table S2. Peripheral blood samples obtained from 8 patients without cGVHD after HSCT were used as controls. The levels of IL-39 (RapidBio, USA) and CXCL13 (R&D Systems, UK) in serum samples were detected using an enzyme-linked immunosorbent assay (ELISA).

### Real-time quantitative polymerase chain reaction (qPCR)

Murine CD4^+^T cells, CD8^+^T cells, and B cells were sorted from splenocytes of C57BL/6 mice, whereas human CD4^+^T cells, CD8^+^T cells, and B cells were sorted from PBMCs of healthy individuals using CD4^+^T, CD8^+^T, and B Cell Isolation Kits according to the manufacturer’s protocol (StemCell Technologies, Vancouver, Canada). Similarly, CD11b^+^ cells were sorted from splenocytes of C57BL/6 mice or human PBMCs using CD11b micromagnetic beads, according to the manufacturer’s protocol (Miltenyi Biotec, Germany). For T cell activation, plates were coated with 2 mg/ml anti-CD3 and 0.4 mg/ml anti-CD28 Abs (BioLegend, San Diego, CA) overnight. 2 × 10^5^ CD4^+^T and CD8^+^T cells (2 × 10^5^) were cultured with various concentrations of mouse rIL-39 for 72 h at 37 °C in a cell culture incubator containing 5% CO_2_. For B cell and CD11b^+^ activation, 2 × 10^5^ B cells and CD11b^+^ cells were cultured with LPS (5 ng/ml) and various concentrations of rIL-39 protein for 72 h. Consistently, activated CD4^+^T, CD8^+^T, B, and CD11b^+^ cells isolated from the PBMCs of healthy individuals were cultured with various concentrations of human rIL-39 for 72 h. Total RNA was isolated using TRIzol Reagent (Invitrogen, Carlsbad, CA, USA), according to the manufacturer’s instructions. cDNA was synthesized using reverse transcription, random hexamer primers, and 10 mM dNTP (Promega). The expression of EBi3 and IL-23p19 in the spleen at 10 days and in the liver, lung, and intestine at 30 days post-HSCT was regarded as control. The relative expression of genes in one organ at the remaining time point was calculated by 2^−ΔΔCt^ according to the control. The primer sequences are listed in Table S1.

### Western blotting analysis

Murine CD3^+^T cells were sorted from splenocytes of C57BL/6 mice, and human CD3^+^T cells were sorted from peripheral blood mononuclear cells (PBMCs) of healthy individuals using a T Cell Isolation Kit, according to the manufacturer’s protocol (StemCell Technologies, Vancouver, Canada). For T cell activation, plates were coated with 2 mg/ml anti-CD3 and 0.4 mg/ml anti-CD28 Abs (BioLegend, San Diego, CA) overnight. Activated T cells were cultured with various concentrations of mouse or human rIL-39 protein for 72 h. Equal amounts of protein were subjected to 10% SDS-PAGE and transferred to a polyvinylidene fluoride (PVDF) membrane. After blocking with 3% BSA, the membrane was incubated with primary antibodies, including STAT1 (D1K9Y), Phospho-STAT1 (58D6), STAT3 (D3Z2G), Phospho-STAT3 (D3A7), and GAPDH (D4C6R) (CST, USA) at 4 °C overnight, followed by incubation with secondary antibodies (Absin, China) at room temperature for 2 h. All results were normalized to GAPDH expression, which was used as the loading control.

### Statistical analysis

Data were presented using GraphPad Prism 7 software (GraphPad Software, San Diego, CA, USA). The nonparametric Mann–Whitney U test was used to analyze the body weight and clinical scores between the groups. Comparisons between two groups were performed using unpaired two-way Student’s t-tests. The diagnostic value of biomarkers was evaluated using ROC curves. Data are expressed as the mean ± SD or SEM. For all statistics, if *P* < 0.05, they were considered statistically significant (*), less than 0.01 or 0.001 were shown as ** or ***, respectively.

## Supplementary Information

**Additional file 1**: **Table S1.** Sequences of primers used. **Table S2.** Clinical characteristics of the patients. **Table S3**. Antibodies used in flow cytometry. **Table S4**. Primary data of CXCL13 and IL-39 concentrations in patients. **Figure S1.** Relative expression of IL-23p19 and EBi3 in spleen and target tissues of cGVHD mice. Irradiated BALB/c recipients were infused with 1×10^7^ bone marrow cells and 1×10^6^ splenocytes from the C57BL/6 mice. Mice infused with bone marrow cells were used as the controls. The expression of IL-39 in the lungs, liver, small intestine, and spleen of recipients was quantified by qPCR on days 30, 40, and 50 after transplantation (n=3, each group and time point). Data are representative of at least three independent experiments. Values are presented as mean ± SEM. **P< 0.01, ***P< 0.001. **Figure S2**: Survival of transduced flag-tagged IL-39 mice in scleroderma and lupus-like cGVHD models. Scleroderma-like (A) and lupus-like (B) cGVHD models were established. The survival of mice was observed for 56 days. Survival was assessed using the Kaplan-Meier method and compared using the log-rank test. **Figure S3**: IL-39 promotes the activation of T cells in the lupus-like cGVHD mice model. Irradiated BALB/c recipients were infused with 5×10^6^ bone marrow cells and 4×10^7^ CD25- splenocytes from the DBA/2 mice. Splenocytes (n=6 per group) were collected and stained for FACS analysis 8 weeks post transplantation. The percentages and numbers of CD4^+^T, CD69^+^CD4^+^T, CD8^+^T, and CD69^+^CD8^+^T cells in lymphocytes from the spleens of the recipients are shown (A). Lymphocytes were isolated from the spleens of recipients and treated with PMA, brefeldin A, and ionomycin for 4-6h. The percentage and number of TNF-α-(B), IL-4-(C) positive T cells and Tregs (D) in the spleens of recipients are shown. Values are presented as mean ± SD. *P< 0.05. **Figure S4**. Anti-IL-39 antibody suppressed secretion of the pro-inflammatory cytokines in vitro. CD3^+^T cells were isolated from the spleens of C57BL/6 mice using magnetic bead sorting. T cells were stimulated with anti-CD3 and anti-CD28 and then treated with PBS, rIL-39, or anti-IL-39 antibody for 72h. The percentage of cytokines in the CD3^+^T cells is shown. Data are representative of at least three independent experiments. Values are presented as mean ± SD. *P< 0.05. **Figure S5**: Effect of IL-39 blockade on scleroderma-like cGVHD development. Irradiated BALB/c recipients (n=4 each group) were infused with 1×10^7^ bone marrow cells and 1×10^6^ splenocytes from the C57BL/6 mice. Fourteen days after transplantation, each mouse in the antibody group received 100μl (100μg) of IL-39 antibody, while each mouse in the control group received 100μl (100μg) of isotype control antibody via intraperitoneal injection twice a week for 6 weeks. The overall survival (A), body weight (B) and GVHD scores (C) are shown. Values are presented as mean ± SEM. *P< 0.05; ***P< 0.001. **Figure S6:** Effect of IL-39 blockade on cGVHD development and immune cells in the target tissues. Irradiated BALB/c recipients (n=4 each group) were infused with 1×10^7^ bone marrow cells and 1×10^6^ splenocytes from C57BL/6 mice. Fourteen days after transplantation, each mouse in the antibody group received 100μl (100μg) of IL-39 antibody, while each mouse in the control group received 100μl (100μg) of isotype control antibody via intraperitoneal injection twice a week for 6 weeks. The percentage and number of CD8^+^TNF-α^+^cells and CD4^+^IL-4^+^ T cells in the intestine (A), the numbers of CD69^+^CD4^+^T cells and CD69^+^CD8^+^T cells in the liver (B) and the percentage and number and of CD8^+^T cells in the lungs (C) are shown. Values are presented as mean ± SEM. **Figure S7**: Phosphorylation of STAT1 and total STAT1 was detected by western blotting in sorted primary T cells from the mice. Plates were coated with 2 mg/ml anti-CD3 and 0.4 mg/ml anti-CD28 Abs overnight. T cells (2×10^5^ T cells were cultured with various concentrations of recombinant mouse IL-39 proteins for 72h. The phosphorylation of STAT1 and total STAT1 detected by western blotting in sorted primary T cells from mice is shown. The data are representative of three independent experiments. **Figure S8**: Effects of IL-39 blockade on IFN-γ and IL-17A expression in CD4^+^ and CD8^+^ donor T cells in the spleens of cGVHD mice. Irradiated BALB/c recipients (n=4 in each group) were infused with 1×10^7^ bone marrow cells and 1×10^6^ splenocytes from the C57BL/6 mice. Fourteen days after transplantation, each mouse in the antibody group received 100μl (100μg) of IL-39 antibody, whereas each mouse in the control group received 100μl (100μg) of isotype control antibody via intraperitoneal injection twice a week for 6 weeks. The percentages and numbers of donor Th1(A), Tc1 (B), Th17 (C), and Tc17 (D) cells in the spleen on day 56 are shown. Values are presented as mean ± SD. **Figure S9**: Effects of IL-39 overexpression on donor CD4^+^ and CD8^+^T cells in the spleens of cGVHD mice four weeks post-transplantation. Irradiated BALB/c recipients were infused with 1×10^7^ bone marrow cells and 1×10^6^ splenocytes from the C57BL/6 mice. Splenocytes (n=4 in each group) were collected and stained for FACS analysis four weeks after transplantation. The percentages and numbers of CD4^+^T, CD69^+^CD4^+^T, CD8^+^T, and CD69^+^CD8^+^T cells in CD3^+^ cells from the spleens of the recipients are shown (A). Lymphocytes were isolated from the spleens of recipients and treated with PMA, brefeldin A, and ionomycin for 4-6h. The percentage and number of TNF-α (B) and IL-4 (C) positive T cells in the spleens of recipients are shown.

## Data Availability

The datasets used and/or analyzed during the current study are available from the corresponding author upon reasonable request.

## Abbreviations

aGVHD: Acute graft-versus-host disease
allo-HSCT: Allogeneic hematopoietic stem cell transplantation
ACS: Acute coronary syndrome
BAFF: B cell activation factor
BFA: Brefeldin A
cGVHD: Chronic graft-versus-host disease
DP: Double positive
ELISA: Enzyme-linked immunosorbent assay
GC: Germinal center
HSCT: Hematopoietic stem cell transplantation
HGT: Hydrodynamic gene transfer
PBMCs: Peripheral blood mononuclear cells
PMA: Phorbol-12-myristate-13-acetate
SP: Single positive
SPF: Specific pathogen-free
TBI: Total body irradiation
Tfh: T follicular helper
