# Supplementary material for: IL-39 promotes chronic graft-versus-host disease by increasing T and B Cell pathogenicity
Source: Exp Hematol Oncol. 2022 Jun 2;11:34. doi: 10.1186/s40164-022-00286-x (PMC9161463; doi:10.1186/s40164-022-00286-x)
Supplement: Supplementary file 1 — Additional file 1: Table S1. Sequences of primers used. Table S2. Clinical characteristics of the patients. Table S3. Antibodies used in flow cytometry. Table S4. Primary data of CXCL13 and IL-39 concentrations in patients. Figure S1. Relative expression of IL-23p19 and EBi3 in spleen and target tissues of cGVHD mice. Irradiated BALB/c recipients were infused with 1×10^7^ bone marrow cells and 1×10^6^ splenocytes from the C57BL/6 mice. Mice infused with bone marrow cells were used as the controls. The expression of IL-39 in the lungs, liver, small intestine, and spleen of recipients was quantified by qPCR on days 30, 40, and 50 after transplantation (n=3, each group and time point). Data are representative of at least three independent experiments. Values are presented as mean ± SEM. **P< 0.01, ***P< 0.001. Figure S2: Survival of transduced flag-tagged IL-39 mice in scleroderma and lupus-like cGVHD models. Scleroderma-like (A) and lupus-like (B) cGVHD models were established. The survival of mice was observed for 56 days. Survival was assessed using the Kaplan-Meier method and compared using the log-rank test. Figure S3: IL-39 promotes the activation of T cells in the lupus-like cGVHD mice model. Irradiated BALB/c recipients were infused with 5×10^6^ bone marrow cells and 4×10^7^ CD25- splenocytes from the DBA/2 mice. Splenocytes (n=6 per group) were collected and stained for FACS analysis 8 weeks post transplantation. The percentages and numbers of CD4+T, CD69+CD4+T, CD8+T, and CD69+CD8+T cells in lymphocytes from the spleens of the recipients are shown (A). Lymphocytes were isolated from the spleens of recipients and treated with PMA, brefeldin A, and ionomycin for 4-6h. The percentage and number of TNF-α-(B), IL-4-(C) positive T cells and Tregs (D) in the spleens of recipients are shown. Values are presented as mean ± SD. *P< 0.05. Figure S4. Anti-IL-39 antibody suppressed secretion of the pro-inflammatory cytokines in vitro. CD3+T cells were isolated from the spleens of [file 40164_2022_286_MOESM1_ESM.pdf]

Table S1: The sequence of primer

Gene	Primer sequence
IL-23p19 (mouse)	Forward: 5’-GGCAGAGATTCCACAAGGACT-3’
	Reverse: 5’-CAGCCATCTCCACACTGGAT-3’
EBi3 (mouse)	Forward: 5’-CATTGCCACTTACAGGCTCG-3’
	Reverse: 5’-TGCAGTGACATTTAGCATGTAGG-3’
IL-23R (mouse)	Forward: 5’-TTCAGATGGGCATGAATGTTTCT-3’
	Reverse: 5’-CCAAATCCGAGCTGTTGTTCTAT-3’
gp130 (mouse)	Forward: 5’-TTCAGATGGGCATGAATGTTTCT-3’
	Reverse: 5’-CCAAATCCGAGCTGTTGTTCTAT-3’
GAPDH (mouse)	Forward: 5’-CTCTGAGTCCTTGAAGGCGTAC-3’
	Reverse: 5’-CCATTCTGGTCGTCCACAGGAA-3’
IL-23p19 (human)	Forward: 5’-CTCAGGGACAACAGTCAGTTC-3’
	Reverse: 5’-ACAGGGCTATCAGGGAGCA-3’
EBi3 (human)	Forward: 5’-TCATTGCCACGTACAGGCTC-3’
	Reverse: 5’-GGGTCGGGCTTGATGATGTG-3’
IL-23R (human)	Forward: 5’-CAGGTCACTATTCAATGGGATGC-3’
	Reverse: 5’-GCAGTTCTTAATTGCTGCTTGG-3’

gp130 (human)	Forward: 5’-CCCCTCAGCAATGTTGTTTGT -3’
	Reverse: 5’-CTCCGGGACTGCTAACTGG-3’
GAPDH (human)	Forward: 5’-AGAAGGTGGTGAAGCAGGCATC-3’
	Reverse: 5’-CGGCATCGAAGGTGGAAGAGTG-3’

Table S2: Clinical characteristics of patients

	Percentage（%）
Number of Patients	50
Age (median, range) (years)	36(16-68)
Sex	
Female	12(24.0)
Male	38(76.0)
Diagnosis	
Acute lymphoblastic leukemia	20(40.0)
Acute myeloid leukemia	15(30.0)
Chronic myeloid leukemia	1(2.0)
Myelodysplastic syndrome	5(10.0)
Mixed phenotype acute leukemia	2(4.0)
T-cell lymphoblastic lymphoma	3(6.0)
Others	4(8.0)
Conditioning regimen	
Modified Bu/Cy	46(92.0)
TBI/Cy	2(4.0)
FBA	2(4.0)
Donor type	
Sibling	24(48.0)

MUD	4(8.0)
Haplo	22(44.0)
ATG	
Yes	26(52.0)
No	24(48.0)
aGVHD	
Yes	21(42.0)
No	29(58.0)
cGVHD	
No	6(12.0)
Mild	11(22.0)
Moderate/Severe	33(66.0)

Abbreviations: Bu/Cy: busulfan/ cyclophosphamide; TBI: total body irradiation; FBA: fludarabine+busulfan+cytarabine; ATG: anti-thymocyte globulin; MUD: matched unrelated donor; aGVHD: acute Graft-versus-Host Disease; cGVHD: chronic Graft-versus-Host Disease.

Table S3: Antibodies in flow cytometry

antibody	clone	company
Mouse, BV650-H-2^b	AF6-88.5	BD Bioscience
Mouse, PE-CF594-CD3e	145-2C11	BD Bioscience
Mouse, BV510-CD4	RM4-5	BD Bioscience
Mouse, PE/Cyanine7-B220	RA3-6B2	BD Bioscience
Mouse, BV421-GL-7	GL-7	BD Bioscience
Mouse, BV510-CD95	Fas, Jo2	BD Bioscience
Mouse, PE-CD4	RM4-5	Biolegend

Mouse, Pacific Blue-CD8	Gl-1	Biolegend
Mouse, FITC-CD69	H1.2F3	Biolegend
Mouse, PE/Cyanine7-TNF-α	MP6-XT22	Biolegend
Mouse, PE-Foxp3	MF-14	Biolegend
Mouse, Allophycocyanin-IL-4	11B11	Biolegend
Mouse, Allophycocyanin-CD185	CXCR5, L138D7	Biolegend
Mouse, Allophycocyanin/Cy7-CD25	3C7	Biolegend
Mouse, PE/Cyanine7-PD-1	29F.1A12	Biolegend

Table S4: The primary data of CXCL13 and IL-39 concentration in patients

**Concentration(pg/ml)**	**CXCL13**			**IL-39**		
	**No**	**Mild**	**M/S**	**No**	**Mild**	**M/S**
	73.33	499.90	778.84	21.18	139.84	60.74
	45.74	88.09	893.84	14.84	55.85	229.20
	107.90	260.20	393.84	82.71	51.46	186.72
	108.68	537.97	144.03	19.72	90.52	80.76
	52.74	217.46	565.95	39.74	84.66	33.39
	292.83	223.68	239.22	41.21	176.95	81.25
		232.22	312.26		16.30	95.41
		586.54	351.49		48.53	80.27
		113.73	260.97		50.48	47.06
		447.06	568.67		47.06	244.83
		103.63	234.17		33.88	44.62
			652.19			168.16
			318.47			252.64
			86.93			123.24
			156.08			38.28

			576.82			45.11
			92.36			113.96
			685.60			75.39
			955.22			173.05
			199.98			73.43
			556.23			54.88
			189.49			53.41
			180.17			226.27
			310.31			38.28
			622.28			170.12
			137.82			245.31
			439.68			221.39
			185.99			93.94
			98.19			167.19
			109.46			49.51
			230.28			47.06
			230.28			49.51
			405.11			33.39
	**Mean**					
	113.50	301.00	368.60	36.57	72.32	112.10
	**SEM**					
	37.47	55.37	42.04	10.25	14.46	12.95

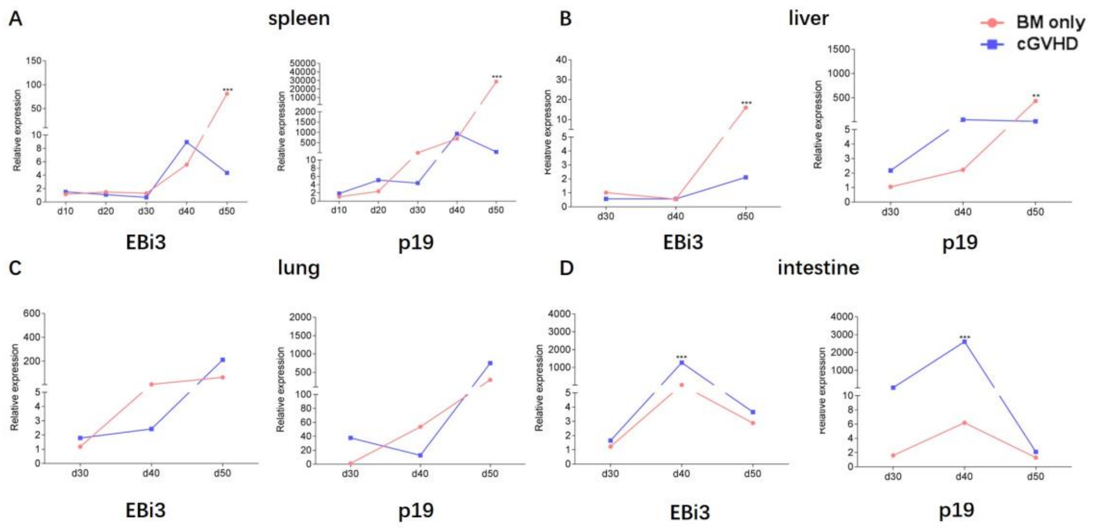

Figure S1: Relative expression of IL-23p19 and EBi3 in spleen and target tissues of cGVHD mice. Irradiated BALB/c recipients were infused with 1×10^7 bone marrow cells and 1×10^6 splenocytes from the C57BL/6 mice. Mice infused with bone marrow cells were used as the controls. The expression of IL-39 in the lungs, liver, small intestine, and spleen of recipients was quantified by qPCR on days 30, 40, and 50 after transplantation (n=3, each group and time point). Data are representative of at least three independent experiments. Values are presented as mean ± SEM. $^{**}P< 0.01$, $^{***}P< 0.001$.

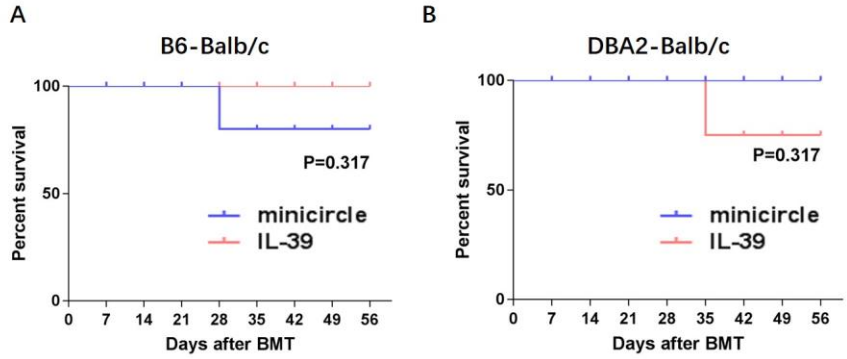

Figure S2: Survival of transduced flag-tagged IL-39 mice in scleroderma and lupus-like cGVHD models. Scleroderma-like (A) and lupus-like (B) cGVHD models were established. The survival of mice was observed for 56 days. Survival was assessed using the Kaplan-Meier method and compared using the log-rank test.

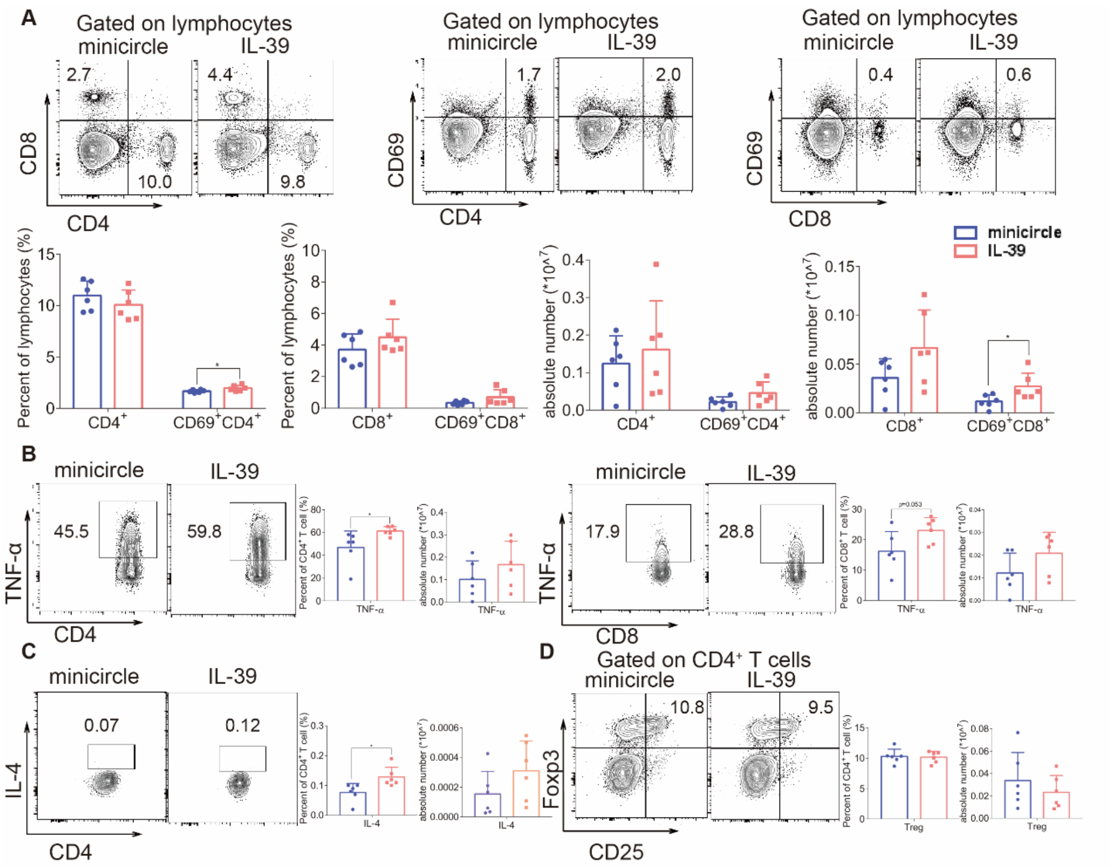

Figure S3: IL-39 promotes the activation of T cells in the lupus-like cGVHD mice model. Irradiated BALB/c recipients were infused with 5×10^6 bone marrow cells and 4×10^7 $CD25^-$ splenocytes from the DBA/2 mice. Splenocytes (n=6 per group) were collected and stained for FACS analysis 8 weeks post transplantation. The percentages and numbers of $CD4^+$T, $CD69^+CD4^+$T, $CD8^+$T, and $CD69^+CD8^+$T cells in lymphocytes from the spleens of the recipients are shown (A). Lymphocytes were isolated from the spleens of recipients and treated with PMA, brefeldin A, and ionomycin for 4-6h. The percentage and number of TNF-α-(B), IL-4-(C) positive T cells and Tregs (D) in the spleens of recipients are shown. Values are presented as mean ± SD. *P< 0.05.

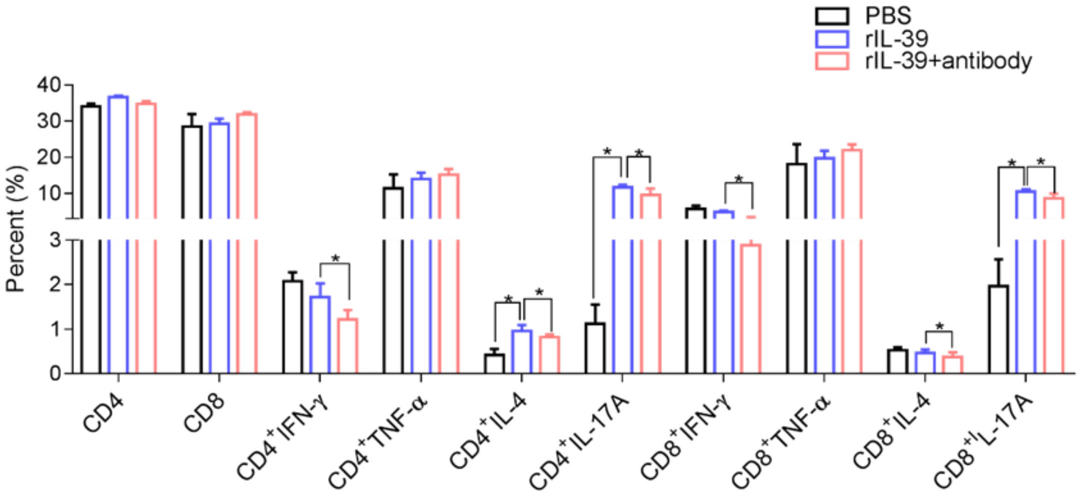

Figure S4: Anti-IL-39 antibody suppressed secretion of the pro-inflammatory cytokines in *vitro*. CD3$^+$T cells were isolated from the spleen of C57BL/6 mice by magnetic bead sorting. T cells were stimulated with anti-CD3/anti-CD28, and then treated with PBS, rIL-39 or anti-IL-39 antibody for 72h. The percent of cytokines in CD3$^+$T cells were shown. Data are the representatives of at least three independent experiments. Values are presented as mean ± SD. *$P< 0.05$.

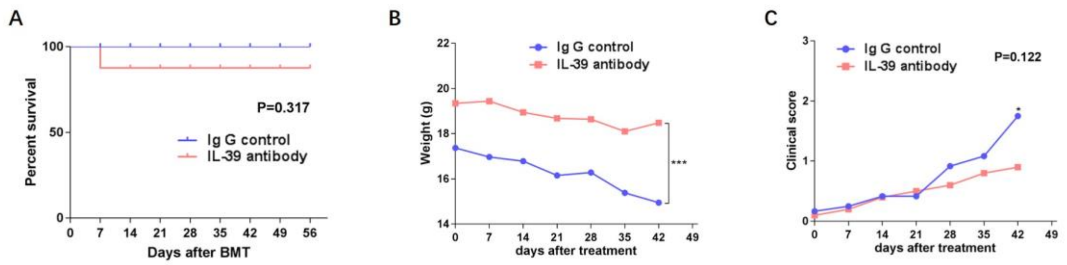

Figure S5: Effect of IL-39 blockade on scleroderma-like cGVHD development. Irradiated BALB/c recipients (n=4 each group) were infused with 1×10^7 bone marrow cells and 1×10^6 splenocytes from the C57BL/6 mice. Fourteen days after transplantation, each mouse in the antibody group received 100μl (100μg) of IL-39 antibody, while each mouse in the control group received 100μl (100μg) of isotype control antibody via intraperitoneal injection twice a week for 6 weeks. The overall survival (A), body weight (B) and GVHD scores (C) are shown. Values are presented as mean ± SEM. *$P< 0.05$, ***$P< 0.001$.

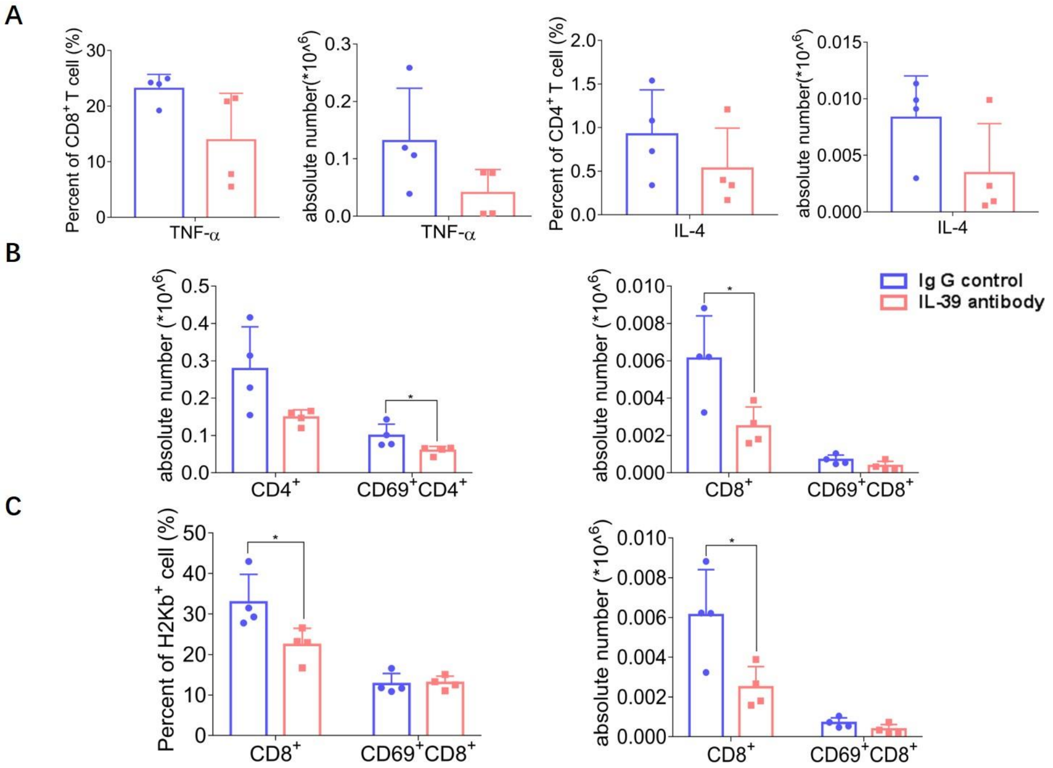

Figure S6: Effect of IL-39 blockade on cGVHD development and immune cells in the target tissues. Irradiated BALB/c recipients (n=4 each group) were infused with 1×10^7 bone marrow cells and 1×10^6 splenocytes from C57BL/6 mice. Fourteen days after transplantation, each mouse in the antibody group received 100μl (100μg) of IL-39 antibody, while each mouse in the control group received 100μl (100μg) of isotype control antibody via intraperitoneal injection twice a week for 6 weeks. The percentage and number of $CD8^+TNF\text{-}\alpha^+$cells and $CD4^+IL\text{-}4^+$ T cells in the intestine (A), the numbers of $CD69^+CD4^+$T cells and $CD69^+CD8^+$T cells in the liver (B) and the percentage and number and of $CD8^+$T cells in the lungs (C) are shown. Values are presented as mean ± SEM. *P< 0.05.

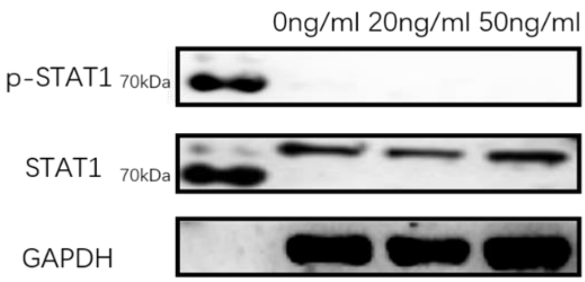

Figure S7: Phosphorylation of STAT1 and total STAT1 was detected by western blotting in sorted primary T cells from the mice. Plates were coated with 2 mg/ml anti-CD3 and 0.4 mg/ml anti-CD28 Abs overnight. T cells (2×10^5 T cells were cultured with various concentrations of recombinant mouse IL-39 proteins for 72h. The phosphorylation of STAT1 and total STAT1 detected by western blotting in sorted primary T cells from mice is shown. The data are representative of three independent experiments.

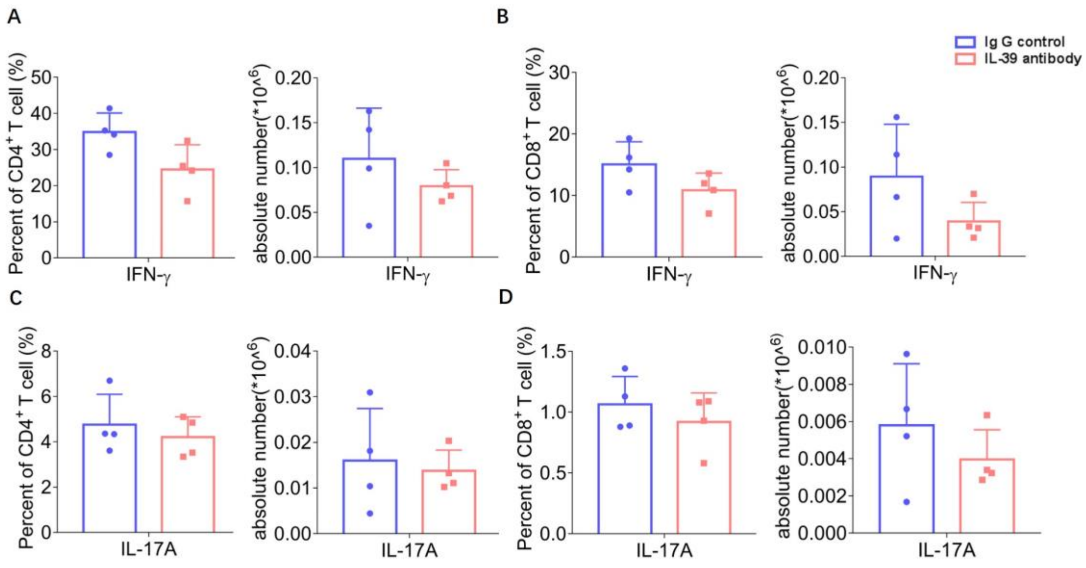

Figure S8: Effects of IL-39 blockade on IFN-γ and IL-17A expression in CD4^{+} and CD8^{+} donor T cells in the spleens of cGVHD mice. Irradiated BALB/c recipients (n=4 in each group) were infused with 1×10^{7} bone marrow cells and 1×10^{6} splenocytes from the C57BL/6 mice. Fourteen days after transplantation, each mouse in the antibody group received 100μl (100μg) of IL-39 antibody, whereas each mouse in the control group received 100μl (100μg) of isotype control antibody via intraperitoneal injection twice a week for 6 weeks. The percentages and numbers of donor Th1(A), Tc1 (B), Th17 (C), and Tc17 (D) cells in the spleen on day 56 are shown. Values are presented as mean ± SD.

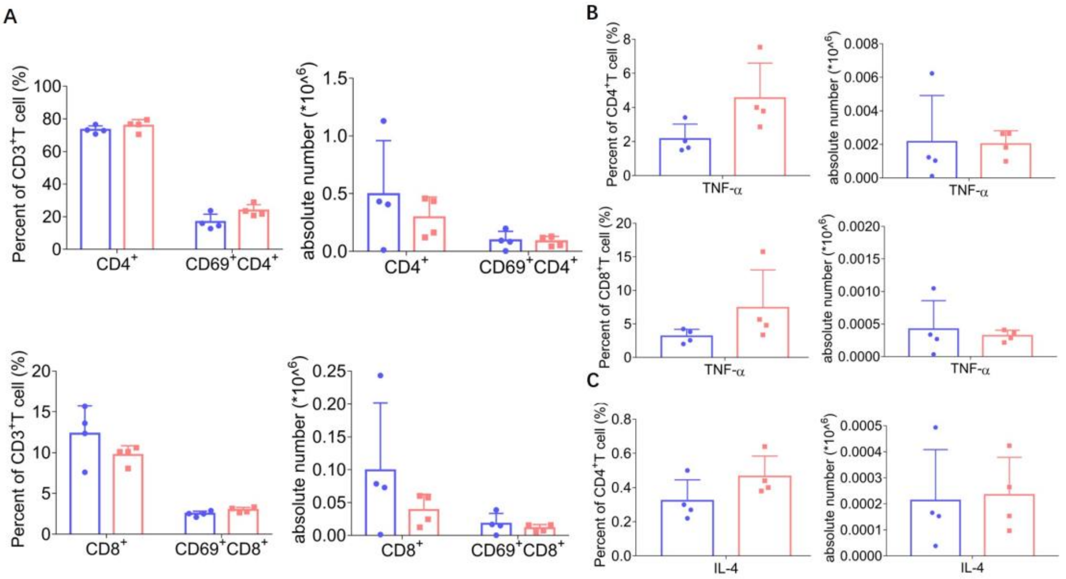

Figure S9: Effects of IL-39 overexpression on donor CD4$^+$ and CD8$^+$T cells in the spleens of cGVHD mice four weeks post-transplantation. Irradiated BALB/c recipients were infused with 1×10^7 bone marrow cells and 1×10^6 splenocytes from the C57BL/6 mice. Splenocytes (n=4 in each group) were collected and stained for FACS analysis four weeks after transplantation. The percentages and numbers of CD4$^+$T, CD69$^+$CD4$^+$T, CD8$^+$T, and CD69$^+$CD8$^+$T cells in CD3$^+$ cells from the spleens of the recipients are shown (A). Lymphocytes were isolated from the spleens of recipients and treated with PMA, brefeldin A, and ionomycin for 4-6h. The percentage and number of TNF-α (B) and IL-4 (C) positive T cells in the spleens of recipients are shown.
